# Challenges and Opportunities in Consumer Neuroergonomics

**DOI:** 10.3389/fnrgo.2021.606646

**Published:** 2021-03-05

**Authors:** Anne-Marie Brouwer

**Affiliations:** TNO The Netherlands Organisation for Applied Scientific Research, Soesterberg, Netherlands

**Keywords:** neuroergonomics, psychophysiology, affective computing, brain-computer interfaces, human-machine systems, mental state monitoring, wearables

## Introduction

As described in the Field Grand Challenge article of this journal (Dehais et al., 2020a), Neuroergonomics is all about understanding the brain at work and in everyday life. Understanding the brain in everyday life is essential for clinical, psychological, and social neuroscience as fundamental research fields, as well as for harnessing neuroscience knowledge for applications, which has been at the core interest of this field from the beginning (Parasuraman, 2003).

In Consumer Neuroergonomics, we focus on the application—products and services that benefit the everyday consumer, in her or his professional capacity or in free time.

The field of Consumer Neuroergonomics can be understood in two ways. One concerns developing and validating neuroscientific consumer goods. For purchase and use by the everyday consumer, these goods need to be not only useful or fun, but also cheap, easy and comfortable to use. The other concerns the application of neuroscientific methodologies to study the user in her/his capacity of a consumer, examining user experience, product usability and product marketing.

With respect to Consumer Neuroergonomics goods, a broad range of neuroergonomic studies work toward their development. Examples are studies on predicting missing auditory alerts (Dehais et al., 2014), predicting memorized visual information (Brouwer et al., 2017b), detecting workload, fatigue and mind wandering (Borghini et al., 2014), usually with the aim of adapting semi-automated systems to better fit the current state of the user (Putze et al., 2018; Dehais et al., 2020b; Roy et al., 2020). Another example is monitoring group attention and engagement using wearable technology for possible use in educational settings (Dikker et al., 2017; Stuldreher et al., 2020; Van Beers et al., 2020). In parallel with scientific work toward developing neuroergonomic applications, an industry emerged providing products and services that (claim to) relate physiological measures to mental state, and that give advice or feedback based on these. Examples of products in this industry are wrist and headbands to monitor and reduce own stress levels, or gadgets to detect mood for entertaining purposes. A trending subfield in scientific studies and industry are tools to modulate brain activity directly through neurostimulation (Tyler et al., 2017; Vosskuhl et al., 2018).

Consumer Neuroergonomics as a discipline that uses neuroscience to study individuals in their capacity as consumers encompasses neuromarketing (Lee et al., 2007; Ariely and Berns, 2010; Stasi et al., 2018), neuroeconomics (Sanfey et al., 2006; Clithero et al., 2008), and consumer neuroscience (Yoon et al., 2012; Plassmann et al., 2015). This application area aims to better understand consumers and their interaction with products and services beyond the traditional self-report surveys and articulated responses from focus groups. Examples are studies on neuroscientific indicators of willingness to pay (Ramsøy et al., 2018) and purchasing behavior (Çakir et al., 2018); using neuroscience to investigate the role of emotions in decision-making (Rampl et al., 2016) and to evaluate advertising or marketing campaigns (Cartocci et al., 2017; Krampe et al., 2018). Similar to Consumer Neuroergonomics as a discipline that develops neuroscientific consumer goods, for Consumer Neuroergonomics as a discipline that studies the consumer, there is already a neuromarketing industry that commercializes this discipline.

Industry and commercial developments represent an essential element of Consumer Neuroergonomics, in that they directly target actual use, taking into account user comfort and user interests. On the other hand, Consumer Neuroergonomics as a science plays a role in the independent assessment of the validity of these products, and drive their improvement.

Below I present drivers that leverage Consumer Neuroergonomics and challenges that, within the broader field of Neuroergonomics, are quite specific for Consumer Neuroergonomic studies. These challenges relate to bridging the gap between science and application, to methodological issues, and to ethics.

## Drivers

There is a great interest in applying information from (neuro)physiological signals. Compared to other research and technology domains, applied neuroscience gets a large share of attention in the media, from students and start-ups as well as larger companies. Three important drivers of this interest can be identified.

Firstly, there is a need in the outside world for accessible methods and technology that can probe mental state through (neuro)physiological sensing in everyday consumers. We ever more often interact with increasingly complex technology throughout the day, both in our personal and professional lives. While we can process a certain amount of information provided by technology, the bandwidth of information from us toward the technology is quite limited, prohibiting optimal adjustment from the technology to the user. Besides an increase in interaction with technology, spurring the need for information about the human to the system, globalization and centralization increases human-human interaction mediated through technology. Especially now during the COVID epidemic, real life meetings related to work or leisure are being replaced by virtual meetings. While this has large advantages e.g. from an environmental point of view, it also drastically reduces the continuous source of information about partners' mental states, such as interest or confusion, that is normally available. Neuroergonomics tools may help to reduce this problem. Another, different need for accessible methods and technology that can probe mental state, directly relates to Consumer Neuroergonomics as a discipline that studies the consumer. It is formulated by developers of products that have reached a level of maturity or quality such that usual tests and questionnaires can no longer predict market success. The hope is that alternative methods, probing mental state in a different way, still can (Köster and Mojet, 2015; Venkatraman et al., 2015; Kaneko et al., 2018). A final example of the need for technology that can monitor mental state is that of monitoring and predicting stress-related problems. Traditional interventions have been found to have positive, but only small effect sizes (Panagioti et al., 2017; Dreison et al., 2018)—neuroergonomic tools may support and expand these traditional approaches in designing timely and personalized interventions.

Besides a drive coming from the need, there is also the drive from technology. There is a huge development of relatively cheap, wearable sensors to measure brain signals, heart rate, and electrodermal activity, often together with different other types of information such as temperature and movement (Michard, 2017; Radüntz and Meffert, 2019; Stojanova et al., 2019; Witt et al., 2019; Masè et al., 2020; Sawangjai et al., 2020). Also, it is possible to extract physiological information from camera images such as heart rate, breathing as well as behavioral information like eye gaze, posture and facial expression (van der Kooij and Naber, 2019; Kong et al., 2021). This enables collecting information about an individual sitting at a laptop without any interference and without requiring any special equipment. These developments open up an unprecedented range of possibilities.

In alignment with technological developments, and fed by (sometimes oversimplified) stories in the popular media and by companies, the third driver is a strong belief in part of the general public that mental state or thoughts can currently be inferred with great accuracy and precision from neurophysiolocal signals. Wrist bands that indicate stress level, and emotion as indicated by lit up brain areas are seen as more “true” than what individuals report to feel. Neurophysiological measures and the addition of irrelevant neuroscience information can unjustifiably lend credence to conclusions and explanations (Canli and Amin, 2002; Farah, 2002; Weisberg et al., 2008; Howard-Jones, 2009). Here, the science of Consumer Neuroergonomics has an important role to play as also discussed below.

## Challenges

Important current challenges in Neuroergonomics as a whole include improving wearable sensors, dealing with artifacts, dealing with limited spatiotemporal resolution neuroimaging, improving generalization across users and context and finding (other) ways to minimize the requirement of training or calibration data (Brouwer et al., 2015; Lotte et al., 2018; Dehais et al., 2020a). Clearly, meeting these challenges are essential for Consumer Neuroergonomics as a subfield as well.

The core of Neuroergonomics, but of Consumer Neuroergonomics in particular, is the integration between fundamental science and its application; between scientists, developers and users. The focus on the benefit of current, and near-future applications calls for close collaboration between (potential) users, engineers, designers and scientists; identification and exploration of suitable application areas; and solid validation where we could focus more on validation in terms of the beneficial effects that we are ultimately interested in. The immediate connection with potential large scale use by laymen, with recording from users rather than only laboratory participants, and dealing with technology that aims to detect mental state without having to ask but therewith also blurring the boundary of consent, also brings ethical challenges. These application-related and ethical challenges are outlined below.

Neuroergonomics research is multidisciplinary by nature, involving research fields ranging from machine learning to psychology. Bringing high-level expertise together on all these areas can be challenging and requires a good team. However, especially important for Consumer Neuroergonomics is the collaboration between scientists, developers, companies and consumers, as well as interdisciplinarity in expertise of different possible sources of information, besides the brain. Since Consumer Neuroergonomics is focused on applications in the relative short term, it is important to know the needs and wishes of the users, and the practical boundaries in the context of (envisioned) use. See for an example of how to implement this Derks et al. (2019), who describe the development of a biofeedback app using a User Centered Design framework and a cyclic developmental process involving the different user groups. With respect to practical boundaries in the context of use, multi-sensor brain measurements or collecting extensive amounts of calibration data may be prohibited due to the burden put on the user. Utilizing other physiological signals, as well as various kinds of information that can be obtained unobtrusively from e.g., a (web)camera, speech and movement can improve interpretation of other (brain) measures, as well as serve as the desired, multifaceted information itself (e.g., Brouwer et al., 2017a; Sargent et al., 2020). There is still a scarcity of studies that computationally combine different types of signals in another way than treating them as features in a model.

While this and other papers identified general areas of Consumer Neuroergonomics applications, e.g., enhancing man-machine teaming and evaluation of products without having to rely only on explicit measures, more specific cases could and should be explored where it is clear that the needs in the application area inherently match the strengths of Consumer Neuroergonomics. Promising application areas are those that clearly gain from continuous, implicit measures since easy alternatives (e.g., self-report or overt performance measures) are not available or usable, and that build on relatively easy interpretable signals. Also, such applications should be envisioned to be useful within the inherent limitations of limited accuracy, obtrusiveness of sensors, calibration data and noise in the context of use. Some examples in this respect include identification of attended speakers through around-the-ear EEG to improve hearing aids (Mirkovic et al., 2016), predicting aggressive outbursts of patients in mental health institutions based on physiology recorded through a wristband (De Looff et al., 2019), and predicting upcoming head rotation through EEG to improve video streaming in Head Mounted Displays (Brouwer et al., 2018). Another promising and timely area may be in awareness training on implicit biased judgement of others, or, as discussed in the beginning, recovering social cues that are lost when moving from real to virtual meetings. The latter may range from indicating where a meeting's attendee is gazing at, to monitoring shared attention through interindividual synchrony in heart rate as recorded through the webcam (cf. Stuldreher et al., 2020). In the context of evaluating consumers' mental state when interacting with systems or products, Consumer Neuroergonomics is especially relevant when mental state is studied over the course of interaction and repeated questioning would influence the mental state of interest itself (Ayaz et al., 2012; Shewokis et al., 2017; Brouwer et al., 2019), or when systematic response biases are expected, e.g., because consumers under study systematically differ in cultural background (Torrico et al., 2018, 2019; Kaneko et al., in press).

With developing applications comes testing and validation. For applications that monitor mental state, e.g., stress, this is usually done by comparing the mental state as estimated by the application to the state as reported by the individual, or by comparing the estimates between different conditions that are assumed to differentially influence mental state of interest. However, for many (envisioned) applications we are not interested in estimating a certain mental state in itself, but in a means to change individuals' behavior, improve individuals' performance or general well-being. We want to do this beyond what is possible with current methods, or in an easier way than currently used methods. Thus, in order to know whether an application works, it is important to connect results as directly as possible to what we are ultimately interested in. From the research perspective, this can be considered as a blessing and a curse. On the one hand, this approach stays clear of the issue of defining mental states, of which it is often unsure how or whether they can be mapped on certain physiological signals (Cacioppo and Tassinary, 1990), and that are muddled and confounded in real life situations. Additionally, it is difficult to retrieve independent, “ground truth” measures of these mental states to train models and validate results, especially when the motivation of the neuroergonomic research or application is to circumvent the use of self-report measures (Brouwer et al., 2020). Finally, for many types of mental states, it is unknown what the “appropriate level” is, i.e., the level corresponding to high performance and high well-being. On the other hand, focusing on performance measures of (ultimate) interest rather than mental state, is difficult since these can be hard to obtain (e.g., success in the marketplace, or pilot's noticing a very rare combination of events that can cause a fatal air traffic accident). Obtaining self-reports on the mental state that is believed to be related to the ultimate goal is easy to do.

The vicinity of Consumer Neuroergonomics to large scale use outside the scientific or clinical sphere generates imminent ethical challenges. One class of ethical challenges lies in a range of issues that comes with monitoring of individuals out of the context of ethically approved experiments, where the aim of the monitoring is to extract information from individuals without them being (continuously) aware of this. While the purpose of this is to not burden the user, or to derive information that is beneficial but otherwise is difficult to obtain, this brings the requirement to treat these private data with care and with consent of the individuals monitored. Ienca et al. (2018) express their concern in this respect and propose safeguarding legal regulations. They also argue for legislation for non-invasive neurostimulation devices, that pose special health risks to the user. Mecacci and Haselager (2019) propose a framework, or a list of criteria, along which ethicists, policy makers and other stakeholders can systematically evaluate (upcoming) “brain reading” technology from an ethical perspective. Criteria include weighing the relevance of the information for the purpose at hand (do we really need this information), and whether the method could be used unknown to the individual. When developing neuroergonomic products, criteria such as these should be taken into account, and possible problems alleviated, from the start.

Another class of ethical challenges lies in the communication with the public and maintenance of high scientific standards. As Neuroergonomics scientists, we should take care that shortened explanations and interpretations of our results do not mislead the general public. This is an especially important role in Consumer Neuroergonomics, where the validity of certain neuroergonomic tools and services cannot always readily be known or judged, and neurotechnology companies may oversell their products (Wexler and Thibault, 2018). Wexler and colleagues argue that this class of ethical issues is the most prominent: “.. the problem with consumer EEG devices is not that the data they gather are rich, accurate, and revealing; the problem is that consumer EEG companies are misleading consumers into thinking it is revealing” (Wexler, 2019).

It is clear that Consumer Neuroergonomics scientists have an important role to play in assisting commercial parties, professionals and consumers in interpreting findings and products related to Consumer Neuroergonomics, both for outlining its possibilities and opportunities to enhance performance and well-being, as well as its (current) limitations, e.g., with respect to generalization across situations. Helping interpret findings and products is also essential to contribute and to shape the public discussion on the possible dangers associated with ethical concerns concerning neuroergonomic applications, ranging from privacy issues to possible consequences of overreliance on neuroergonomic applications. We should note that high scientific standards do not imply that research has to follow common practice as originated from traditional laboratory research. It is, especially in this field, important to go beyond the strictly controlled laboratory experiments, and to explore the sensitivity of the various innovative sensors and techniques under multiple types of daily life conditions. However, we should be transparent on the downsides this may bring, discuss, and examine possible influence of sensor noise and confounds, and be careful in interpreting the meaning of the findings, especially in terms of what this means for applications. Rayatdoost et al. (2020a,b) follow an approach that is sound from an applied, scientific and ethical view. They investigate classification of emotion based on signals recorded by EEG sensors, show and discuss that the major part of classification success can be attributed to muscle artifacts, and subsequently use these ‘artifacts' as information to improve their classification. They thus show that EEG sensors can be useful to classify emotions (especially in cases that the face is hidden under a Head Mounted Display), but do not claim that this information is obtained from the brain.

## Final Remarks

In sum, Consumer Neuroergonomics can flourish through benefits that Neuroergonomics can bring, through a push from technology and interest of the public. Studies and products that would propel Consumer Neuroergonomics involve potential users and expertise from different fields; they harness, if needed, multiple modalities; they are targeted to solve a well-circumscribed problem that is hard to solve in other (cheaper or simpler) ways; they show that the neuroergonomic product or approach actually benefits the user; they respect the user's safety and privacy; and they are transparent on the functioning and limitations of the product or approach.

With this paper, I hope to have acknowledged existing challenges and at the same time illustrated the opportunities in this exciting new field. Opportunities, open questions and enthusiasm are also manifested in the Frontiers in Consumer Neuroergonomics Research Topics that are already in place. There is no doubt that the establishment of Frontiers Neuroergonomics will help to bring the field forward.

## Author Contributions

The author confirms being the sole contributor of this work and has approved it for publication.

## Conflict of Interest

The author declares that the research was conducted in the absence of any commercial or financial relationships that could be construed as a potential conflict of interest.
